# New Drugs. Appliances, and Things Medical

**Published:** 1892-04-16

**Authors:** 


### NEW DRUGS, APPLIANCES, AND THINGS MEDICAL.

[All preparations, appliances, novelties, etc., of which a notice is desired, should be sent for The Editor, to care of The Manager, 140, Strand, London, W.C.]

### MALT PASTILLES.

(Rowntree and Co., York.)

The above well-known firm have forwarded to us specimens of their Malt Pastilles. These are well made pastilles, in shape and appearance like the ordinary sweetmeat, but on examination we find that the firm have ingeniously combined in them the advantage of (1) a large amount of malt extract; (2) a considerable quantity of diastatic ferment; (3) a very pleasant form of sweetmeat; (4) and an absence of alcohol. The pastilles form a really delicious sweet, which can be taken by anyone, and which, instead of hindering digestion, will aid it. We should imagine that for children they would greatly assist in the administration of cod liver oil, as a bribe for the taking of it, and as a digestive when it has been taken. We think Messrs. Rowntree have earned the thanks of the medical profession by producing this elegant preparation.

### ÆSCULAP WATER.

Among old-established mineral waters Æsculap holds a high place. Recent analysis shows that its quality remains constant, and clinical experience proves that its efficacy and suitability are unimpaired. As a mild saline, fitted alike for men, women, and children, Æsculap has few equals and no superior. The taste of the water is so little disagreeable that many children will prefer it to almost any other aperient. The recent investigations of Dr. Alassy go to prove that Æsculap has an important function in the prevention of stone in the bladder. For this purpose its use should be more or less constant. Bitter salines of this character are of very great service to the dyspeptic, the constipated, and the depressed in spirits. We have pleasure in again speaking well of Æsculap Water.

